# Modeling Input Factors in Second Language Acquisition of the English Article Construction

**DOI:** 10.3389/fpsyg.2021.653258

**Published:** 2021-04-22

**Authors:** Helen Zhao, Jason Fan

**Affiliations:** ^1^School of Languages and Linguistics, University of Melbourne, Melbourne, VIC, Australia; ^2^Language Testing Research Centre, University of Melbourne, Melbourne, VIC, Australia

**Keywords:** competition model, frequency, reliability, English article construction, second language, structural equation modeling, corpus, language production

## Abstract

Based on the Competition Model, the current study investigated how cue availability and cue reliability as two important input factors influenced second language (L2) learners' cue learning of the English article construction. Written corpus data of university-level Chinese-L1 learners of English were sampled for a comparison of English majors and non-English majors who demonstrated two levels of L2 competence in English article usage. The path model analysis in structural equation modeling was utilized to investigate the relationship between the input factors and L2 usage (frequency and accuracy of article cue production). The findings contribute novel and scarce empirical evidence that confirms a central claim of the Competition Model, i.e., the changing importance of cue availability and cue reliability in the frequency and accuracy of production. Cue availability was found to determine L2 production frequency regardless of level of L2 competence. Cue reliability was the input factor that differentiated competence levels. When learners stayed at a relatively lower L2 proficiency, cue reliability played an important role in influencing L2 frequency of usage rather than accuracy of usage. When learners developed increased exposure to and stronger competence in the target language, cue reliability played a significant role in determining learners' success of cue learning. The study is methodologically innovative and expands the empirical applicability of the Competition Model to the domain of second language production and construction learning.

## Introduction

The current study is the first corpus-based study that statistically models the contributions of the input variables of *cue availability and cue reliability* to second language (L2) acquisition of the English article construction. The study is guided by the theoretical framework of the Competition Model (MacWhinney, 1987, 2012, 2017), which views language as a system of form-function mappings. Forms (e.g., *the*) serve as *cues* for the activation of functions (e.g., *uniqueness*). We represent an article cue in the form of “article function | article form,” following the convention of the model that represents form-function mappings as “X | Y” (the interpretation X given a cue Y). Cues differ in their inherent properties, the two most important of which are availability and reliability. Cue availability is the proportion of times the cue is available over the times it is needed, whereas cue reliability is the proportion of times the cue leads to the intended interpretation over the times it is available (MacWhinney, 2017). In another word, availability is related to the cue's frequency of usage, whereas reliability reflects the contingency of cue(form)-function association. Some cues (e.g., “*second mention* | *the*,” such as *I bought a book. The book is so interesting*) express frequent functions and demonstrate a reliable form-function association. Some cues (e.g., “*river* | *the*,” such as *the Mississippi River*) are related to infrequent functions and thus have low availability, but they always correctly predict the use of the form and hence have high reliability.

Decades of empirical research on the Competition Model have provided strong evidence supporting the importance of the input variables in determining the outcomes in L1 and L2 acquisition (see MacWhinney, 1997, 2001 for a review). Cue reliability has consistently been found to be the most important predictor for cue strength in sentence processing experiments with L1 adults. Most of these experiments adopted a simple sentence interpretation procedure that asks participants to judge sentences with conflicting cues. Adult L2 learners were observed to begin with a reliance on the cues with the highest cue strength in the L1, and to gradually change to target-like cue strength settings as learners' L2 competence increased.

Thus far there is a dearth of studies that have applied the Competition Model to explaining acquisition data obtained from production tasks. And there is a lack of descriptions about several issues including the relationship between the input variables, to what extent the input variables contribute to explaining L2 learning outcomes, and how this contribution might change as a function of learners' L2 development. Therefore, the present study attempted to address these research gaps through exploring the influence of input properties on L2 acquisition of form-function mappings in the English article construction. A written corpus on Chinese learners of English, a learner group that has been widely known to have experienced challenges in acquiring English articles (Master, 1997; Robertson, 2000), was sampled and coded with a usage-based article cue system (Zhao and MacWhinney, 2018) for a structural equation modeling analysis on variable relationships among input variables (cue availability and cue reliability) and L2 variables (frequency and accuracy of L2 usage). Due to the lack of longitudinal data in the corpus, two groups (English majors and non-English majors) were sampled for a cross-sectional comparison on learners with different levels of L2 competence with regard to the influence of the examined input factors.

## The Competition Model

The Competition Model (MacWhinney, 1987, 1997, 2008; Bates and MacWhinney, 1989) presents a functionalist account for language structure, processing, and acquisition. Functionalism is the belief that “the forms of natural languages are created, governed, constrained, acquired and used in the service of communicative functions” (MacWhinney et al., 1984, pp. 128). *Forms* are the external phonological and word order patterns that are used in words and syntactic constructions, whereas *functions* are the communicative intentions or meanings that underlie language usage (MacWhinney, 1997). For instance, the form of the word *cat* is the set of phonological cues that contain the sound sequence /*kAt*/. The functions for this word involve the expression of the various semantic properties of the animal, along with its visual and auditory images. Lexical items and syntactic constructions can be understood in terms of form-to-function mappings. One-to-one mappings between form and function are rare in natural languages, which are composed primarily of many-to-many relationships. The pressure of communicative function, operating in accord with the constraints of neurolinguistic processing, is considered to be the primary determinant of language development, processing, and evolution.

In the Competition Model, forms serve as *cues* to activate meaning (or function). This principle applies to both language comprehension and production. The model views the comprehension of a sentence as the outcome of the interpretation given the formal cues, whereas the model views the production of a sentence as the outcome of a competition between many alternative forms of expression. The past morpheme -*ed* cues the interpretation of the simple past. Some French noun endings such as -*sion* and -*ité* activate the feminine interpretation of grammatical gender, whereas some endings such as -*aire* and -*isme* cue the masculine assignment. Due to the polysemous nature of language (many-to-many mappings), formal features are often not reliable cues for a particular meaning interpretation in language comprehension. For example, -*s* in English is associated with multiple functional markings including plural, third person singular present, and possessive. The information value carried by the morpheme -*s* is light, since speakers need additional contextual information to achieve accurate functional reading. Meanwhile, unreliable cues may not be favored by language speakers during production, as there can be other alternative forms that are readily available for usage and can express the same meaning or fulfill the same function.

The major predictive construct in the Competition Model is *cue validity*. Cue validity is “the information value of a given linguistic device as a cue to an underlying meaning or intention” (Bates and MacWhinney, 1989, pp. 37). The single most common interpretation of cue validity is in terms of the conditional probability that an event X will occur given a cue Y, that is, p (X | Y). With this property, we can quantify the degree to which that a formal feature informs its associated function. Cue validity can be measured in samples of spoken or written language such as conversational input data available from the CHILDES corpora (MacWhinney, 2000) or text counts of researcher self-composed corpus that represents target language use in the task domain (McDonald and MacWhinney, 1989; MacWhinney, 2017). The value of cue validity yielded by the corpus counts is used to generate predictions for sentence interpretation and for cue-driven language acquisition. Forms that are computed to be of a high conditional probability should win over the competition with forms of a lower conditional probability; forms of a high conditional probability should be acquired early and be the strongest determinants of processing in adults.

Cue validity is composed of three components (Bates and MacWhinney, 1989): *cue availability, cue reliability*, and *conflict reliability*. Cue availability “represents the extent to which a cue is there when you need it” and is measured numerically “as the ratio of the cases in which the cue is available over the total number of cases in a task domain” (Bates and MacWhinney, 1989, pp. 41). For example, the availability of the cue of the definite article (*the*) to indicate second mention is very high in English, but is relatively low when *the* is used to indicate absolute uniqueness (e.g., *the moon, the earth*). This is simply because second mention is a more frequent function than absolute uniqueness. The availability of the cue “*second mention* | *the*” can be computed as the ratio of its frequency of occurrence over the total number of article usage in a given spoken or written language sample. All things being equal, cues related to a frequent function will be acquired earlier than cues related to infrequent functions.

Cue reliability is another important component of cue validity. Reliability is “the degree to which a cue leads to the correct interpretation when you count on it” and is computed numerically “as a ratio of the cases in which a cue leads to the correct conclusion over the number of cases in which it is available” (Bates and MacWhinney, 1989, pp. 41). For instance, the reliability of the cue “*absolute uniqueness* | *the*” is very high. Because when this cue is present, it always correctly predicts the use of the definite article. In contrast, the reliability of the cue “*singular countable with post-modifiers* | *the*” (e.g., *the man she is dating*) is relatively low, as there are many cases when a singular countable noun requires the indefinite article despite being post-modified (e.g., *a man she is dating*, assuming that she may be dating more than one man).

Conflict reliability is a special kind of reliability. Specifically, it is the reliability of a cue when it competes directly with other cues. For example, case marking conflicts with word order in a sentence such as “*the dogs saw she*.” In English, word order “wins” the competition and the sentence is given an SVO interpretation, whereas in Dutch, the same sentence is resolved in favor of case marking and is given an OVS interpretation. Such conflicts between word order and case marking are rare even in Dutch. But the English article construction is rich with cue conflicts and thus conflict reliability is an important property that influences article acquisition. The above example of the cue “*singular countable with post-modifiers* | *the*” illustrates conflict reliability, as there are two alternative competing forms (*the man she is dating* vs. *a man she is dating*) associated with the same functional feature (post-modified singular countability). Thus, this cue is not high in conflict reliability. In contrast, another cue “*non-countable with post-modifiers* | *the*” (such as *the land they own* and *the perfume in the bottle*) has high conflict reliability, given that we cannot say *land they own* or *perfume in the bottle*. In such cases, the cue that supports choice of the definite article dominates over the cueing of zero by non-countability. Some competing alternatives in the article system are simply an outcome of conventionality. For example, names of buildings, bridges, theaters, hotels etc. generally take the definite article, as in *the Babel Building, the Sydney Harbor Bridge, the Majestic Theater, the Peninsula Hotel* and *the British Museum*. But there are also such proper names that take the zero, such as in *Rockefeller Center, Buckingham Palace, London Bridge*, and *Grand Hyatt*. In addition, British English accepts *the High Street* and *the Main Street*, while in American English street names are generally used without the definite article (Radden and Dirven, 2007).

The English article construction allows a lot of co-existing alternative forms like this. Cues such as the singular countable nouns or non-countable nouns with post-modifiers offer more analyzable properties, as further cueing from overall discourse patterns can then support the choice of one of the options over the other. The proper name cues are not analyzable because of the idiosyncrasy in their usage. Conflict reliabilities of article cues can vary a lot, thus making it harder for learners to acquire but providing a good test ground for predictions about the model-based input properties.

In the Competition Model, language acquisition is characterized as input-driven learning. The model describes language speakers' (learners') linguistic representations “in terms of a complex set of weighted form-function mappings, a dynamic knowledge base that is constantly subject to change” (Bates and MacWhinney, 1989, pp. 13). This dynamic knowledge is termed *cue strength*. Under ideal conditions, the value of cue strength converges on the value of cue validity. Consequently, the order of importance of cues to meaning for adult speakers should closely reflect cue validity estimates. This has been consistently confirmed in Competition Model experiments in which cues are set in conflict with each other (see MacWhinney, 1997, for a review). In such experiments with the same paradigm of a sentence interpretation task (untimed or timed), participants are presented with a series of simple transitive sentences (e.g., *the dogs saw she*) composed of two concrete nouns and a transitive action verb and are asked to judge which of the nouns is the agent. The sentence stimuli always include competing and/or converging cues to sentence meaning. Participants' agent identification reveals which cue(s) they rely on in sentence interpretation, on the basis of which researchers can determine the relative order of cue strength assigned by the speaker. According to MacWhinney (2017), this basic sentence interpretation method has been robustly applied “in 52 empirical studies involving 18 different languages” and the model has also been tested with more online processing and neuroimaging methods including “self-paced reading, eye-movement monitoring, ERP, fMRI, and cross-modal priming methods” in more recent studies (p. 291).

Changes in cue strength in the course of language development have been tied to cue validity. An important prediction of the Competition Model is that L1 and L2 acquisition is controlled primarily by cue availability at the early stage, followed by a lengthy phase of learning controlled by overall cue validity (cue reliability becoming more important than cue availability), with the ultimate phase of learning dominated by conflict validity as learners fine-tune the form-function mappings in relatively less frequent situations that involve cue competition (Bates and MacWhinney, 1989; McDonald and MacWhinney, 1991; MacWhinney, 1997, 2008, 2017). Learners of beginning proficiency in a language heavily rely on high-frequency cues readily available to them for comprehension and production. As more cues are acquired, cue strengths change and begin to mirror cue validity as assessed only over those sentences involving conflict between these cues (McDonald and MacWhinney, 1989). Importantly, learners adjust their cue strengths when they make interpretation errors and receive feedback. The ultimate stage of language acquisition involves learners' successful resolution of cue conflicts in favor of target-like cue-outcome interpretations. This theoretical prediction has been well-supported by empirical evidence in L1 acquisitional studies (e.g., McDonald, 1986), but lacks adequate empirical evidence from L2 studies. MacWhinney (2017) summarizes the empirical findings of the Competition Model on child and adult monolinguals as follows (pp. 293):

Children begin learning to comprehend sentences by first focusing on the most available cue in their language.

As children get older, cue strengths converge on the adult pattern with the most reliable cue growing most in strength.

As children get older, their reaction times gradually get faster in accord with the adult pattern.

Compared to adults, children are relatively more influenced by cue availability, as opposed to cue reliability.

Cue strength in adults and older children (8-10 years) is not related to cue availability (since all cues have been heavily encountered by this time), but rather to cue reliability. In particular, it is a function of conflict reliability, which measures the reliability of a cue when it conflicts directly with other cues.

The existing L2 studies within the Competition Model framework (e.g., McDonald, 1987; Liu et al., 1992; Sasaki, 1994; Su, 2001; Tokowicz and MacWhinney, 2005; Morett and MacWhinney, 2013) have primarily attempted to validate the model's prediction on language transfer, i.e., L2 learners initially rely on cues that are dominant in their L1 in L2 sentence processing and would gradually acquire new cue-strength patterns in the L2 (MacWhinney, 1997, 2012). In other words, L2 learners would transfer their L1-based processing strategy to L2 processing, resulting in non-native processing of the L2 which may (or may not) be replaced by L2 cues as a function of L2 development.

The degree of the adaptation to the target processing strategies differs across studies. This difference may be due to the particular language-specific differences in strategies by native speakers of the two languages, bilingual proficiency (Kilborn and Cooreman, 1987; McDonald and Heilenman, 1991; Rounds and Kanagy, 1998; Su, 2001; Jackson, 2008; Morett and MacWhinney, 2013; Pham and Ebert, 2016), amounts of L2 exposure (McDonald, 1987; Sasaki, 1991; Heilenman and McDonald, 1993), and starting age of acquiring the L2 or age of arrival in the L2 speaking environment (McDonald, 1987; Liu et al., 1992; Reyes and Hernandez, 2006; Pham and Kohnert, 2010). Early bilinguals with a young age of onset of learning the L2 tend to show an amalgamation of processing strategies from both the L1 and the L2, thus demonstrating an “in-between” profile (Hernández et al., 1994). Late adult bilinguals' sentence interpretation strategies tend to show forward transfer (Su, 2001), especially at a lower L2 proficiency or with a limited amount of L2 exposure. They adopt L1-processing strategies in interpreting L2 sentences. With continued exposure and growth of L2 proficiency, adult bilinguals rely increasingly on a coalition of L1 and L2 cues in processing L1 and L2 sentences, thus showing cue weight adjustment and sometimes backward transfer.

There is a dearth of L2 studies that test the model's prediction on the changing weight of cue availability and cue reliability at different stages of L2 learning (Comeaux and McDonald, 2018). In addition, all the above studies have tested the model's prediction by collecting language processing data. However, the Competition Model applies to language production as well. The model views the production of each sentence as the outcome of a competition between many alternative forms of expression. Which form to choose in production also depends on cue availability (how frequent the cue is readily available for use) and cue reliability (the conditional probability of being able to use the form whenever you have the idea) (MacWhinney, 1997). Language production is perhaps the area that has the most urgent need for more empirical data in order to test some core predictions of the Competition Model as a general language learning model not restricted to comprehension.

## A Usage-Based Account of the English Article Construction

The English article construction provides a good testing ground for the influence of input properties. The articles, despite its seemingly simple formal system (*the, a, an*, Ø), contain a very large collection of functions. Zhao and MacWhinney (2018) put forward a usage-based framework to the analysis of the English article construction in which they analyzed a full range of 86 functional usages of the articles (excluding idiomatic usages such as *by Ø hand*). In this complex space of form-function mappings, there are a large variation among article cues with regard to various input properties including availability, reliability, prototypicality (Ellis and Collins, 2009; Wulff et al., 2009; Zhao and MacWhinney, 2018), and transparency (McDonald and Plauché, 1995). Table 1 lists the ten article cues with the highest availability according to Zhao and MacWhinney (2018) corpus analysis and their availability and reliability values.

**Table 1:** Properties of the ten article cues with the highest availability (Zhao and MacWhinney, 2018).

	**Article cue**	**Example**	**Availability**	**Reliability**
1	Plural | Ø	Ø Books	0.154	1.000
2	Non–countable | Ø	Ø Water	0.120	1.000
3	Singular countable with post–modifiers | the	*The* man she is dating	0.119	0.435
4	Singular countable | a/an	*A* Shakespearean drama	0.115	0.988
5	Plural with post–modifiers | the	*The* letters I received today	0.063	0.392
6	Part of | the	I'm returning this coat for a refund. *The* zipper broke.	0.056	1.000
7	Second mention with variation | the	I saw a peacock at the zoo. *The* bird was beautiful.	0.043	1.000
8	Second mention | the	I saw a peacock. *The* peacock was beautiful.	0.035	1.000
9	Names of countries, cities or states | Ø	Ø Hong Kong	0.033	0.892
10	Non–countable with post–modifiers | the	*The* wealth of her parents	0.025	0.785

According to the cognitive grammar account of articles by Langacker (1991, 2008), the article cues are not a random list but constitute a grammatical category of nominal predicates that define the figure and ground relationship in discourse. Through nominal grounding devices such as articles and other determiners (e.g., *this, that*), the speaker directs the hearer's attention to the figure (i.e., the intended discourse referent) in relation to a ground (i.e., the speech event and its participants). Prototypical configurations of nominal grounding include type, instance, and definiteness. A nominal type involves an open-ended set of actual or imagined instances, while no instance is being profiled. A prototypical exemplar of the type configuration is the cue “*plural* | Ø” (e.g., Ø* cars*). A type configuration is transformed to an instance configuration through the speaker's profiling of a specific instance in the general type. A prototypical exemplar of an instance configuration is the cue “*singular countable* | *a/an*” (e.g., *a Shakespearean drama*). The type/instance distinction lies in profiling (attention-directing) and in specificity. The instance conception is the default expectation for the indefinite article. Definiteness applies to situations of unique instantiation, i.e., when there is only one unique instance available of the specified type in the immediate scope of the discourse context constructed by knowledge of the speaker and the hearer. Prototypical exemplars of the definiteness configuration include some high-frequency cues with post-modifiers such as “*singular countable with post-modifiers* | *the*” (e.g., *the man she is dating*) or “*plural with post*–*modifiers* | *the*” (e.g., *the letters I received today*) and the second mention cue (*second mention* | *the*, e.g., *I saw a peacock. The peacock was beautiful*).

Langacker (2008) also describes some non-prototypical types of grounding relationship. The zero article used with mass nouns encodes zero grounding due to the semantic nature of the nouns. Count nouns denote objects (e.g., *apple, book*), whereas mass nouns denote substances (e.g., Ø* water, gold*) (Radden and Dirven, 2007).^1^ The conceptual construal of an object type involves well-delineated boundaries between individuated instances of the same type. It is easier for the speaker to pick out one or some of the instances from the type for grounding. Substances by contrast has no inherent boundaries and, as a result, are continuous rather than discrete and individuated. The inherent unboundedness of mass nouns makes it resistant to instantiation for profiling, thus encoding zero grounding relation, unless we impose externally added boundaries with the help of count nouns (e.g., *a glass of water, a piece of gold*).

Grounding can also be intrinsic, which is the configuration for many of the “proper name” cues. Typical examples of proper names are personal names, country and city names, geographic names, institutional names, architectural names, etc. Langacker regards these name cues as the configuration of intrinsic grounding, “since the very meanings of such expressions imply the identifiability of their referents, they do not require a separate grounding element” (Langacker, 2008, p. 272). Therefore, proper names should be inherently definite. However, the article usages of the English proper name cues are highly idiosyncratic (Radden and Dirven, 2007; Verspoor and Huong, 2008; Zhao and MacWhinney, 2018). For example, English lake names usually take the zero article (e.g., *Lake Michigan*), whereas river names usually take the definite article (e.g., *the Mississippi River*). Many English park names use the zero article (e.g., *Central Park*), whereas many garden names use the definite article (e.g., *the New York Botanical Garden*). The article usages of English proper names in general follow historical conventions and demonstrate high idiosyncrasy and low transparency in terms of the selection of article forms. Among the 86 article cues identified in Zhao and MacWhinney (2018), the proper name cues occupy a large type space, but only a small proportion of token frequency.

## The Present Study

The above review of the literature suggests that the Competition Model is a robust psycholinguistic model of input-based learning which follows its own methodological approach to quantitatively predicting and validating crosslinguistic variations and trajectories of language acquisition in monolingual and multilingual settings. While previous research has provided insights on these issues, a few important gaps have been identified. First, almost all studies of the Competition Model only relied on sentence comprehension processing data. It is true that the model is traditionally known as a sentence processing model. However, the theoretical concepts and assumptions of the model are applicable to language production. To test the validity of the model on production data will significantly expand the theoretical scope of the model and opens up a new empirical direction for the development of the model. Second, the majority of the Competition Model studies have adopted a sentence interpretation task (with variations in its implementations). When we test the model on production data, we need new task designs with methodological innovations. The current study adopts corpus-based naturalistically elicited written production data. Third, previous studies of the Competition Model that involve bilingual speakers have predominantly focused on the investigation of transfer of language processing strategies. Very few studies (McDonald, 1987; McDonald and Plauché, 1995; Comeaux and McDonald, 2018) have tested the model's prediction on the changing weight of cue availability and cue reliability at different stages of language learning. Two of such studies (McDonald and Plauché, 1995; Comeaux and McDonald, 2018) actually examined artificial language learning, which only gives indirect implications to the acquisition of natural languages. The contribution of cue availability and cue reliability at different stages of language learning is a core theoretical value of the model and needs to be tested with more empirical evidence from natural language learning.

The present study aims to investigate how cue availability and cue reliability affect L2 learners' productive use of English article cues. Learners' use of article cues is operationalised as L2 frequency (token frequency) and accuracy of cue usage. The large number of cues in the article system makes it possible to statistically model the relationship between input properties (cue availability and reliability) and learner usage (L2 frequency and accuracy of usage). The written corpus under investigation is based on data collected from college-level Chinese-speaking EFL learners whose L1 does not have an equivalent article system. The corpus does not provide information on learners' English proficiency, which would be a more direct measure of stages of L2 learning. However, the corpus includes data collected from English majors and non-English majors. The two cohorts provide us with a good cross-sectional comparison on levels of L2 competence as a result of their different amounts of target language exposure and language use. Specifically, the study seeks to examine the following research questions:

1. How do input properties of English article cues (cue availability and cue reliability) influence Chinese EFL learners' frequency and accuracy of article cue usage in written production?
2. Do the influences of cue availability and cue reliability on L2 article usage differ according to learners' level of L2 competence?

## Materials and Methods

### Data Source and Coding

Our data are drawn from the written section in the Spoken and Written English Corpus of Chinese Learners (Version 2.0) (SWECCL) (Wen et al., 2008). SWECCL was one of the largest corpora constructed based on data obtained from Chinese-speaking EFL learners. Learner texts in the written section were collected from college students in 34 universities in China mainland. The sampling of the universities has a good coverage of geographic areas and university rankings.

The majority of the written texts are argumentative essays based on prompts (see Appendix 1 for a complete list of the sampled essay prompts). There are two types of written texts, timed and untimed, depending on whether the participants were given time restriction for the written task. Texts were initially collected from learners' handwritten documents and then were manually typed and included into the corpora.

Learner sampling included both English majors and non-English majors. Years 1-4 English-major texts were available in the corpus, whereas only Years 1-2 non-English-major texts were available. For a fair comparison, only Year 1 and Year 2 essays from both majors were sampled for the current study. Approximately 20 texts from the timed essays in the four sub-groups (English-major Year-1, English-major Year-2, Non-English-major Year-1, and Non-English-major Year-2) were randomly sampled. Timed measurements tend to elicit learners' implicit knowledge (Ellis et al., 2009) which is a more reliable measure of learners' L2 competence. Only essays with more than 150 words were selected. Many essays with shorter than 150 words are found to be incomplete and lack a clear essay structure.

It is hard to define or compare the English proficiency levels of the two learner samples. Years 1-2 Non-English majors normally take the College English Test Band 4 (CET4), whereas Years 1-2 English majors are made to take the Test for English Majors Band 4 (TEM4). CET4 and TEM4 are considerably different tests, neither of which has been formally linked to the CEFR levels (Council of Europe, 2001), thus resulting in no direct comparison of the two cohorts' English proficiency. Our experience with these cohorts of English learners suggests that Years 1-2 non-English majors are generally at the B1 level on the CEFR scale (intermediate proficiency), whereas Years 1-2 English majors generally have a B2 level of English proficiency (upper intermediate proficiency).

Four samples with a sum of 16,989 words were generated based on the above criteria (Table 1): English-major Year-1 (4,707 words), English-major Year-2 (5,858 words), Non-English-major Year-1 (3,683 words), and Non-English-major Year-2 (2,741 words). A total of 3,004 noun phrases (NPs) were identified as the obligatory contexts for the use of all types of determiners, including articles and other determiners (quantifiers, possessives, and demonstratives). English majors produced longer texts per essay than non-English majors. Year-2 English majors write longer essays than Year-1 English majors. By contrast, Year-2 non-English majors write much shorter essays than Year-1 non-English majors. This pattern of results applies to all the indexes of NP, determiner, and article productions in the sample (Table 2).

**Table 2:** SWECCL data sample.

	**English majors**		**Non-English majors**	
	**Year 1**	**Year 2**	**Year 1**	**Year 2**
Texts	18	21	19	17
Words	4707	5858	3683	2741
Words per text	261.50	278.95	193.84	161.24
NPs with all determiners	847	1085	626	446
NPs with all determiners per text	47.06	51.67	32.95	26.24
Quantifiers per text	4.50	5.14	2.58	2.12
Possessives per text	4.94	6.57	5.42	4.18
Demonstratives per text	2.06	1.76	1.05	0.82
Obligatory NPs for article use (token)	640	801	454	315
Obligatory NPs for article use per text (token)	35.56	38.14	23.89	18.53
Obligatory NPs for article use (type)	200	234	168	149
Obligatory NPs for article use per text (type)	11.11	11.14	8.84	8.76

In fact, the percentage of non-article determiner use (quantifiers, possessives, and demonstratives) was higher in the sampled learner essays than that of native English academic texts reported in Master (2013). Native English academic writers used a higher percentage of articles in the noun reference system. This suggests that Chinese learners may have “avoided” using articles to a certain extent. This could be attributed to the absence of a comparable article system in their L1 (Li and Thompson, 1981) and that learners from an article-less background often find it difficult to fluently use English articles (Butler, 2002; Ionin et al., 2008). The “overuse” of non-article determiners may be related to L1 transfer (Robertson, 2000), as there is widespread use of determiners in Mandarin Chinese which functions in part to signify definiteness (Li and Thompson, 1981). Zhao and Shirai (in press) provides a detailed account of this phenomenon in their analysis of the same learner corpus sample as the current study.

Two thousand two hundred and ten obligatory NP contexts of article use were identified in the sample. These include correct article suppliance, incorrect omission and incorrect suppliance of the article form. The first author and a trained native English speaking research assistant manually coded the obligatory NP contexts for (a) article cue type and for (b) accuracy of usage in obligatory contexts (SOC). The two coders achieved a high interrater reliability (*k* = 0.86) after discussing and resolving differing codes.

Cue types were coded with the coding scheme consisting of 86 article cues developed by Zhao and MacWhinney (2018). As an example, “*So the children must learn how to compete to protect themselves*.” Here, the use of the definite article *the* is an error since the writer intends it to be a general category of children rather than referring to a specific group of children. Here, *the* was coded as a token of and counted into the accuracy of the cue “*plural* | Ø,” i.e., use Ø with plural nouns unless they are uniquely identifiable.

Certain forms were excluded from analysis. When there are two parallel NPs, both of them were coded when there are no non-article premodifiers such as possessives or quantifiers. For example, in the phrase “a lot of troubles to *college* and *society*”, both “college” and “society” were coded. Both of them were coded as the obligatory contexts for the zero article. But in the phrase “for *your commanders* or *commercial partners*,” only the first NP “your commanders” was coded for the possessive use. The second NP “commercial partners” was excluded from coding, since we cannot judge whether the zero article was used due to the possessive (*your*) or due to the cue “*plural* | Ø.”

We also excluded the erroneous forms that invite ambiguous interpretations. For example, the NP “*foreigner”* in the sentence “I think communicating with *foreigner* is the thing you really want to do” was excluded. The preferred form of the noun “foreigner” in this particular discourse context is its plural form “foreigners.” Therefore, the first interpretation of this error is the omission of the plural marker -*s*. Yet, the singular form of the noun “a foreigner” is also grammatically correct in this sentence, though not preferred. So the error might also be interpreted as an omission error of the indefinite article *a*. Such cases were excluded from coding to avoid ambiguous interpretations. Errors related to misuses of parts of speech were also excluded from coding. For instance, the NP “*independence*” in the sentence “We can learn to be *independence* in universities” was a grammatical error since an adjective (*independent*) rather than a noun is required in the slot. Similarly, we also excluded coding on the adjective “*healthy*” in the sentence “The good *healthy* for them are very important.” Gerunds were also excluded from coding.

We distinguished between *tokens* of article cues, counting all the tokens of an article cue, and *types* of article cues, tallying only one instance of the cue type regardless of the number of tokens that belong to it. For example, if three plural NPs such as *children, schools*, and *companies* were identified in a text, they were coded as three tokens and one type of the cue “*plural* | Ø”. Learners’ L2 frequency of article cue usage for the statistics analysis was calculated with token frequency. To be comparable to the calculation method of cue availability, we used percentage of frequency (i.e., the number of tokens of a cue divided by the total number of article tokens) instead of raw frequency. A learner's accuracy of performance on an article cue was calculated with the suppliance in obligatory context (SOC) analysis, i.e., number of correct suppliances divided by number of obligatory contexts. The SOC analysis was counted with token frequency rather than type frequency.

The availability and reliability of article cues were adopted from the results in Zhao and MacWhinney (2018). In this article, they reported an extraction and validation of a total of 86 cues in the English article system. They also calculated the availability and reliability of these cues in naturally occurring English sentences with a corpus count method (McDonald and MacWhinney, 1989). They constructed a mini-corpus comprised of 38 texts covering 10 common genres of English written texts (academic, encyclopedia, magazine, newspaper, novel, drama, children's story, recipe, etc.) on a wide range of topic areas (politics, economy and finance, education, history, geography, technology, entertainment, sports, travel, food, etc.). The texts were selected from well-known publications to represent native speaker written English. The inclusion of a large variety of written genres and topic areas generates a language sample that is likely to closely mimic what college English learners (including English majors and non-English majors) are experiencing in their English exposure.

### Data Analysis

To investigate the two research questions, a path model analysis in structural equation model (SEM) was implemented in this study. SEM is a powerful statistical technique that can be viewed as a coming together of several statistical models: multiple regression, path analysis and factor analysis (Kunnan, 1998). A systematic review study by In'nami and Koizumi (2011) reveals that SEM has been widely and increasingly utilized in applied linguistics research.

Compared with traditional multivariate procedures such as analysis of variance (ANOVA) or multiple regression analysis, SEM has several salient advantages. In a typical ANOVA or multiple regression analysis, for example, researchers are interested in understanding whether the variance in the dependent variable is accounted for by one or multiple independent variables (Field, 2009); however, it is not easy or even possible to explore the relationships between multiple independent and dependent variables. An SEM analysis, in contrast, is typically implemented to investigate the complex relationships between and among multiple independent and dependent variables. Furthermore, SEM takes a confirmatory, hypothesis-testing approach which means researchers can specify a conceptual model *a priori* which delineates the relationships between multiple variables of interest based on theories or relevant previous research. Next, this conceptual model can be tested against the empirical data.

Another strength with SEM lies in its capability of assessing or correcting for measurement error of variables which traditional analysis procedures are not equipped with, thus enabling researchers to interpret the relationships among variables more accurately by separating measurement error. In addition, whereas traditional multivariate procedures can analyze the direct relationship between variables, SEM is capable of analyzing both the direct and indirect relationships among a certain set of variables. An investigation of the indirect relationship entails the understanding of whether an independent variable affects a dependent variable through a mediating variable (In'nami and Koizumi, 2011). Thanks to these unique strengths of SEM, it was utilized in this study. It should be noted, however, that the path model analysis was implemented in this study because no latent variables were included in our analysis.

The Competition Model predicts input-based cue-driven language acquisition. As indicated by our research questions, we were interested in understanding the relationships between input properties (cue availability and reliability) and learner usage (L2 frequency and accuracy of usage). As such, we predict that cue availability and reliability determine how learners use the article cues in terms of their frequency and accuracy of L2 usage. Cues with higher availability and reliability should be used more frequently and accurately by learners. Furthermore, cue availability is predicted to play a more important role at a relatively lower L2 proficiency, whereas cue reliability is of less consequence at a lower L2 proficiency level but will increase its significance when learners progress to higher competence in L2 usage.

**Figure 1:**
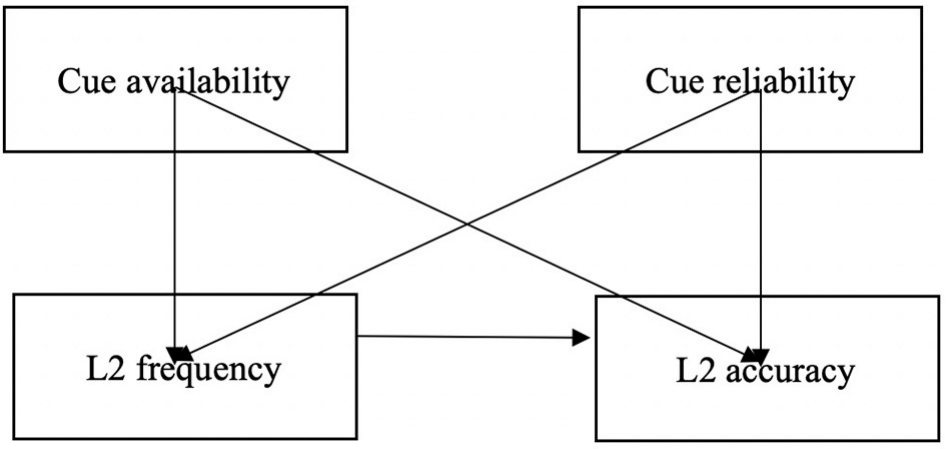
The model with hypothesized relationships between variables.

In view of the Competition Model and previous research, a conceptual model was specified depicting the hypothetical relationships between the four variables of interest (see Figure 1). As illustrated in this model, cue availability and cue reliability are two predictor variables which are hypothesized to affect both L2 frequency and L2 accuracy. In addition, we also predict a relationship between L2 frequency and L2 accuracy because we hypothesize that learners' frequency of using article cues influences their accuracy of usage. This initial model with the relationships that we specified about these four variables was tested against the data that was generated through our coding process. Model fit could be assessed through a number of indices (Kline, 2005; Byrne, 2006). A non-significant Chi-square test, for example, is usually a good indicator that the model fits the data, though the result is sensitive to sample size. In this study, we used the Chi-square test as well as several model fit indices, including the comparative fit index (CFI), the normed fit index (NFI), and the goodness-of-fit index (GFI), all of which should be over 0.90 to indicate satisfactory model-data fit.

After testing this model, another question that was of interest to this study is whether the parameter estimates between the variables of interest were equivalent across the two groups of English-major and non-English major learners. This was explored through testing the same model against the data from the two English learner groups separately and comparing the analysis results. The path model analysis in this study was implemented in EQS 6.3 (Bentler and Wu, 2005).

## Results

### Frequency and Accuracy Distributions

We first report the descriptive statistics of all the sampled learners' (75 texts) frequency and accuracy of using article cues. Results showed that English majors (39 texts) used 40 types of article cues, whereas non-English majors (36 texts) used 35 types of article cues. English majors showed a mean percentage of frequency per cue of 0.03 (SD = 0.05) and a mean accuracy of 0.84 (SD = 0.25). Non-English majors had a mean percentage of frequency per cue of 0.03 (SD = 0.06) and a mean accuracy of 0.82 (SD = 0.28).

Table 3 lists the top six article cues with the highest L2 frequency in the two majors. Apparently, both groups used roughly the same set of cues with the highest availabilities in the English article system (see Table 1), but with a different frequency order. The frequency order among the English majors resembled that of the cue availability order more closely than that of the non-English majors. Despite the resemblance of the ordering, the English majors' percentages of frequency of using the high-frequency cues were a lot higher than their corresponding availabilities. For example, “*plural* | Ø” has an availability of 0.154, but a percentage of frequency of 0.258; “*non*–*countable* | Ø” has an availability of 0.12, but a percentage of frequency of 0.209. These suggest that, though mimicking the overall frequency distribution of article usage in English written texts, English majors relied more heavily on the few top-ranking cues in the frequency list.

**Table 3:** Top six article cues with the highest L2 frequency among English and non-English majors.

	**Article cue**	**Example**	**Percentage of frequency**	**Accuracy**
**English majors**				
1	Plural | Ø	Ø Books	0.258	0.870
2	Non–countable | Ø	Ø Water	0.209	0.912
3	Singular countable | a/an	*A* Shakespearean drama	0.126	0.853
4	Non–countable with post–modifiers | the	*The* wealth of her parents	0.041	0.947
5	Second mention | the	I saw a peacock. *The* peacock was beautiful.	0.038	0.962
6	Singular countable with post–modifiers | the	*The* man she is dating	0.034	0.917
**Non-English majors**				
1	Non–countable | Ø	Ø Water	0.295	0.727
2	Singular countable | a/an	*A* Shakespearean drama	0.185	0.766
3	Plural | Ø	Ø books	0.139	0.854
4	Habitual locations | Ø	Go to Ø school	0.049	0.763
5	Singular countable with post–modifiers | the	*The* man she is dating	0.033	0.962
6	Non–countable with post–modifiers | the	*The* wealth of her parents	0.028	0.909

**Figure 2:**
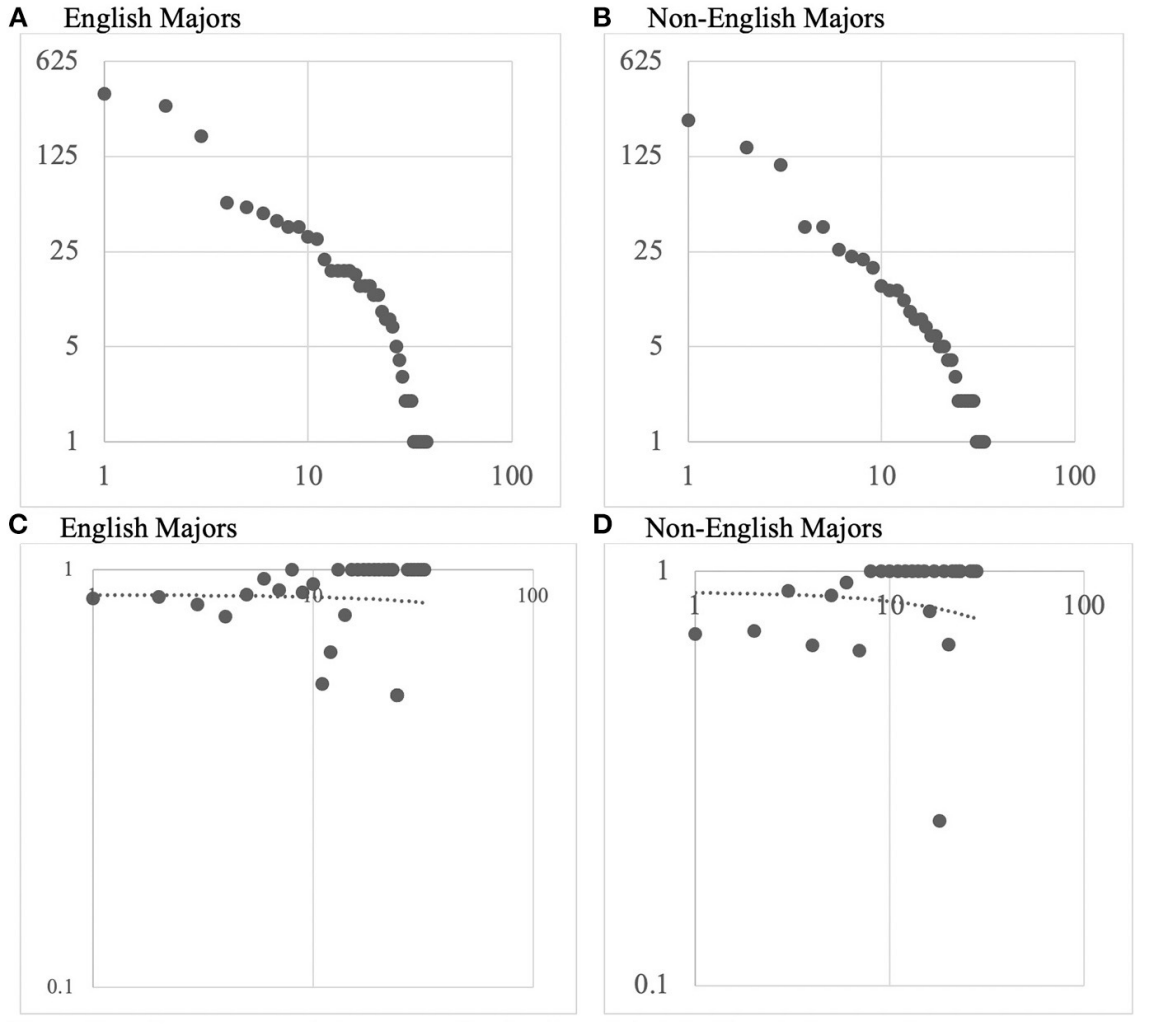
Frequency and accuracy distribution of articles cues in learner groups. **(A,B)**: X axis = Log(token frequency rank); Y axis = Log(token frequency). **(C,D)**: X axis = Log(token frequency rank); Y axis = Log(accuracy).

Results demonstrate that the frequency distribution of article cues in learner texts (Figures 2A,B) is Zipfian (Zipf, 1935), with the most frequent article cues accounting for the majority of all the tokens. Figures 2C,D are log–log plots (with the trendline) that illustrate the accuracy distribution of cues ranked for frequency. The top-ranking cues did not show the highest level of accuracy. A decent number of lower frequency cues obtained a full percentage of accuracy, many of which are definite article cues, such as *ranking words* | *the* (e.g., *the first*), *superlative* | *the* (e.g., *the best one*), *uniqueness* | *the* (e.g., *the Sun*), *anaphoric reference in phrase* | *the* (e.g., *the Harvard faculty*), *specific collectives of people* | *the* (e.g., *the Republican party*), *time of the day/week/season* | *the* (e.g., *in the morning*), *historic periods* | *the* (e.g., *the 1990's*), etc. The overall patterns of frequency and accuracy distributions were the same among the English and non-English majors.

One-way univariate analyses of variance (ANOVAs) revealed a main effect of major in terms of token frequency of article cue production [*F*_(1,71)_ = 62.935, *p* < 0.0001, partial η2 = 0.470], type frequency [*F*_(1,71)_ = 14.065, *p* < 0.0001, partial η2 = 0.165], and accuracy of usage [*F*_(1,71)_ = 5.619, *p* = 0.020, partial η2 = 0.073]. English majors produced significantly more tokens and types of article cues and more accurately than those of non-English majors. Year 2 learners, however, did not outperform Year 1 learners in each major respectively, as the year level was not significant in terms of token frequency [*F*_(1,71)_ = 0.497, *p* = 0.483, partial η2 = 0.007], type frequency [*F*_(1,71)_ = 0.001, *p* = 0.971, partial η2 = 0.000], or accuracy [*F*_(1,71)_ = 0.394, *p* = 0.532, partial η2 = 0.006].

### Modeling the Relationship Between Input Factors and L2 Cue Use

The skewness and kurtosis values for the four variables of interest across the three groups (i.e., English major group, non-English major group, and the whole group) exceed |3.3|, suggesting violation of data normality at the variable level (Field, 2013). For example, the kurtosis values for L2 frequency are 11.35 (all), 11.72 (English major group), and 12.13 (non-English major group) respectively. In addition, Mardia's normalized estimates are 12.71 (all), 12.09 (English majors), and 9.61 (non-English majors), all suggesting multivariate non-normality (Byrne, 2006). Given that data non-normality can affect chi-squares and consequently model-data fit indices based on chi-squares, the maximum likelihood estimation method, the default parameter estimation method in EQS, was not adopted in this study; rather, the robust maximum likelihood method, which produces the Satorra-Bentler corrected Chi-square statistic, was adopted in this study (e.g., Byrne, 2006; In'nami and Koizumi, 2011).

The correlation matrix between these variables across the three groups are presented in Appendix 2. The results indicate that cue availability and L2 frequency are significantly and positively correlated (*p* < 0.01), and the pattern is consistent across all learners together, as well as the subgroups of English majors and non-English majors. Compared with the English majors (*r* = 0.83), the correlation between these two variables in the non-English group appears slightly less strong (*r* = 0.75). For the non-English major group, cue availability and cue reliability are significantly and negatively correlated (*p* < 0.05); the relationship between these two variables, however, is non-significant for the other two groups of English language learners. In what follows, we present the results from modeling the data of the whole group first, and then the two subgroups of English majors and non-English majors.

#### Whole-Group Analysis

The model with hypothetical relationships between variables was tested against the data in EQS 6.1 (Bentler and Wu, 2005). As mentioned previously, since no latent variable was included in the model, a path model analysis, as opposed to a full structural equation modeling analysis, was implemented (Byrne, 2006). Four model-data fit indices were employed to assess whether the model fit the data satisfactorily, including the Chi-square test, CFI, NFI, and GFI. A non-significant Chi-square test is a good indicator that the model fits the data well; in addition, CFI, NFI and GFI should be over 0.90 to indicate satisfactory model-data fit. The results indicate that the model fits the data satisfactorily (S-B Chi-square = 0.21, df = 1, *p* = 0.65; CFI = 1.000, NFI = 0.993, GFI = 0.997). The model with standardized parameter estimates is presented in Table 4.

**Table 4:** Standardized parameter estimates across groups.

**Variables**	**Parameter estimates**		
	**All**	**English majors**	**Non-English majors**
Cue availability → L2 frequency	0.83^**^	0.83^**^	0.79^**^
Cue reliability → L2 frequency	0.15	0.15	0.31^*^
Cue availability → L2 accuracy	0.28	0.24	0.18
Cue reliability → L2 accuracy	0.21	0.31^*^	−0.02
L2 frequency → L2 accuracy	−0.16	−0.16	−0.18

** *p < 0.01*;

* *p < 0.05*.

*R^2^ for L2 frequency is 0.720 (whole group), 0.720 (English major group), and 0.728 (non-English major group); R^2^ for L2 accuracy is 0.063 (whole group), 0.099 (English major group), and 0.016 (non-English major group)*.

As indicated in Table 4, cue availability significantly and positively predicts L2 frequency (*p* < 0.01) suggesting that a higher level of cue availability leads to improvement in L2 frequency. The standardized estimate in this model can be interpreted in the same way as a regression coefficient. The standardized parameter estimate from cue availability to L2 frequency is 0.83, indicating that one standard deviation change in cue availability would lead to 0.83 standard deviation change in L2 frequency. Cue availability also has a positive and non-significant effect on L2 accuracy (β = 0.28, *p* > 0.05). Similarly, cue reliability, the other predictor variable, has a positive and non-significant effect on L2 frequency (β = 0.15) and L2 accuracy (β = 0.21). Finally, L2 frequency has a negative and non-significant effect on L2 accuracy (β = −0.16).

For the two dependent variables in this analysis, that is, L2 frequency and L2 accuracy, the R^2^ for the former is 0.720, indicating that the two predictor variables (i.e., cue availability and cue reliability) explain a considerable proportion of the variance in this variable; the R^2^ for L2 accuracy, on the other hand, is much smaller (0.063), suggesting that a minimal amount of variance is explained by the three independent variables in combination, that is, cue availability, cue reliability, and L2 frequency.

#### Subgroup Analysis

We performed a path model analysis of the data from each learner group. First, we tested the model against the data of the English majors. Results indicate that this model fits the data satisfactorily (Chi-square = 1.000, df = 1, *p* = 0.75, CFI = 1.000, NFI = 0.996, GFI = 0.999). The standardized parameter estimates are presented in Table 4.

Similar to what we found about the whole group, cue availability has a positive and significant effect on L2 frequency (β = 0.83, *p* < 0.01); it also has a positive effect on L2 accuracy (β = 0.24), though the result is not statistically significant. Cue reliability has a positive yet nonsignificant effect on L2 frequency (β = 0.15). Different from what we found about the whole group sample, however, cue reliability has a positive and significant effect on L2 accuracy (β = 0.31, *p* < 0.05) in the English major group. Regarding the relationship between the two dependent variables, L2 frequency has a negative and nonsignificant effect on L2 accuracy (β = −0.16).

The two predictor variables of cue availability and cue reliability explain a considerable proportion of the variance in L2 frequency (R^2^ = 0.720); similar to what we found about the whole sample, the three variables (i.e., cue availability, cue reliability, and L2 frequency) in combination explain only a negligible amount of variance in L2 accuracy (R^2^ = 0.099).

Next, we tested the model against the data from the non-English major group. Results indicate that this model fits the data reasonably well (Chi-square = 2.16, df = 1, *p* = 0.14, CFI = 0.961, NFI = 0.939, GFI = 0.939).

As shown in Table 4, the two predictor variables, that is cue availability and cue reliability, have a positive and significant effect on L2 frequency, though the effect of cue availability appears much stronger (cue availability: β = 0.79, *p* < 0.01; cue reliability: β = 0.31, *p* < 0.05). Cue availability has a positive yet nonsignificant effect on L2 accuracy (β = 0.18). Different from what we found about the English majors, cue reliability has a negligible effect on L2 accuracy (β = −0.02). Finally, L2 frequency has a negative and nonsignificant effect on L2 accuracy (β = −0.18).

Similar to what we found about the English major group, the two variables of cue availability and cue reliability explain a considerable proportion of the variance in L2 frequency (R^2^ = 0.728); the three variables (i.e., cue availability, cue reliability, and L2 frequency), however, explain only a minimal amount of variance in L2 accuracy (R^2^ = 0.016). To facilitate the comparison of research findings, the standardized parameter estimates of each path in the model across the three groups are presented in Table 4.

## Discussion

Out of the complete collection of cues in the English article system, college-level Chinese EFL learners used less than half of the cues in writing academic essays. This could be due to the specific genre of the written corpus. The L2 texts were argumentative essays which may result in a higher portion of certain cue usage. Zhao and MacWhinney (2018) availability list, however, is based on L1 texts of mixed genres (academic texts inclusive). Given the produced cues, learners demonstrated a native-like Zipfian distribution of article cue usage. This was regardless of major (English or non-English) and college year (1st or 2nd). Their frequency of cue usage was strongly influenced by the input property of cue availability. They used roughly the same set of most cues that top the availability ranking, a very small number of which they tended to rely more on than native speakers.

These high-frequency cues are the prototypical exemplars of the type (*plural* | Ø), instance (*singular countable* | *a/an*), and definiteness (*singular countable with post*–*modifiers* | *the*; *non*–*countable with post*–*modifiers* | *the*; *second mention* | *the*) configurations, and of the zero grounding configuration (*non*–*countable* | Ø). Given that there are a large number of cues in the English article system, learners relied on the most frequent ones more heavily than they are available in the input. These high-frequency exemplars play a crucial role in the learners' formation of the schematic configurations of the type, instance, and definiteness grounding relations. Similar to the role of path-breaking verbs such as *give* in the ditransitive construction or *make* in the resultative construction (Goldberg, 1999; Campbell and Tomasello, 2001), the prototypical exemplars in the English article construction guide learners' semantic categorization through their high input frequency and semantic compatibility with the configurations of the nominal grounding relations.

Meanwhile, a large number of idiosyncratic cues were not identified in the sample. Learners did produce tokens for a number of idiosyncratic cues such as *names of countries, cities or states* | Ø (e.g., Ø* Australia*), *historic periods* | *the* (e.g., *the 1990's*), *political and military institution used alone* | *the* (e.g., *the Ministry of Education*), *disease name* | Ø (e.g., Ø* cancer*), *language* | Ø (e.g., Ø* English*), *XX University* | Ø (e.g., Ø* Yale University*), *generic inventions* | *the* (e.g., *the computer*). It is also true that a large number of idiosyncratic cues were not produced. These include many cues that describe geographic features (bodies of water, continental landforms), architectural features (buildings, constructions, halls, malls, stadiums, hotels, theaters, bridges, parks, stations, etc.), street names, music instruments, religion, directional terms (north, south, left, right, top, bottom), etc. These idiosyncratic cues configure intrinsic grounding which is a type of non-prototypical grounding (Radden and Dirven, 2007; Langacker, 2008). Due to their low input frequency (therefore low familiarity to the learner), low prototypicality and high idiosyncrasy in usage, learners might avoid producing unfamiliar idiosyncratic cues in timed written production. Also, many of these cues may require more specific semantic domains of usage, and thus understandably did not appear in the sampled texts that are argumentative essays on topics (Appendix 1) such as the pros and cons of modern technology. Due to a smaller set of cue types in L2 usage, the percentages of frequency of the top-ranking cues became relatively larger than their corresponding availabilities in L1 texts.

Our findings confirmed the previous literature (Ellis and Ferreira-Junior, 2009; Matusevych et al., 2016) that input frequency determines its production frequency in the L2. Cue availability made an equally high contribution to L2 production frequency at the two levels of L2 competence. But the findings also showed that input frequency did not significantly predicts success in production (i.e., accuracy of usage). Instead, accuracy was shown to be influenced by cue reliability, and only in the English major group. Thus, cue reliability was found to be the significant input factor that differentiated levels of L2 competence. This result aligns well with the Competition Model prediction on reliability as the most important predictor for cue strength in sentence processing among adult native speakers (Bates and MacWhinney, 1989; MacWhinney, 1997, 2008, 2017).

The current study's results provide the comparable empirical evidence in L2 production. As discussed earlier, the model views sentence production as the outcome of a competition between many alternative forms of expression. Faced with a writing task under time pressure, L2 writers selected the most readily available cues among the many alternative forms for productive use. Successful usage is determined by cue reliability (the conditional probability of being able to use the form whenever the speaker has the idea to express) (MacWhinney, 1997). As a great part of the reliabilities of article cues involves conflict reliabilities, successful article usage is defined not only by the strengthening of individual cues as a result of increasing input exposure and cue use with feedback, but more importantly by the successful resolution of cue conflicts through a refined distinction between the phrasal, sentential, and discursive contexts associated with the competing cues. The finding that reliability only had a significant influence on accuracy of usage among the English majors and not among the non-English majors indicates that the English majors had surpassed the period of free variation use of competing cues whereas the non-English majors had not.

The ANOVA analysis confirmed that English majors indeed demonstrated stronger competence in all aspects of productive English article usage than non-English majors (i.e., token and type frequency, and accuracy). At an approximately B1 level, non-English majors can produce simple texts on familiar topics and can describe experiences and events and briefly give reasons and explanations for opinions and plans (Council of Europe, 2001). They were predominantly influenced by frequency distributions of cues and heavily relied on high-frequency cues. Learners in this cohort also strongly favored the use of cues with high reliabilities. In a timed essay writing setting, the need to express the message efficiently outweighs the need for expressiveness. They tended to produce simpler and shorter clauses and NPs that are most readily available to them and that they felt most confident in producing.

At an approximately B2 level, English majors can produce clear, detailed texts on a wide range of topics and explain viewpoints on topics giving the advantages and disadvantages of various options (Council of Europe, 2001). They also strived for efficiency but their higher competence in English allowed them to achieve better expressiveness in conveying intended messages. They produced longer essays that contained more details and used a larger range of article cues that accompanied the larger variety of word choices. English majors were strongly influenced by input frequency, and yet they have developed a more fine-tuned awareness toward the complexity of form-function mappings in the article system due to their much more expanded exposure to and intensive training on the English language. They became more knowledgeable about cue competition in the article system. They could allocate more attention to particular elements in discourse (Kilborn and Cooreman, 1987) and to the cueing of article forms by structural and contextual features (Zhao, 2020). These are good evidence suggesting that the learners in our sample with a higher level of L2 competence have shown patterns of article cue usage that approximate the native speaker norm, whereas lower-level learners failed to do so.

An important contribution that the current study aims to make is to test the methodological expansion of the Competition Model to explaining learners' production data. Most previous Competition Model studies tested a very small number of cues (e.g., word order, animacy, subject-verb agreement) in one study and used a variety of the sentence interpretation task to determine cue strength. The current study used written corpus data collected via a free elicitation method (except for the controlled timing during data collection) and the SEM analysis to statistically model the relationships among variables. The quantitative modeling was only possible because of the large number of cues in the article system. Even though learners only produced less than half of the naturally occurring cues, the produced number constituted a decent enough amount for the modeling analysis. Our first endeavor in the current study showed that this new methodological approach in Competition Model testing was feasible and could generate powerful novel findings that previous studies did not show. It is applicable to testing on highly polysemous linguistic structures that contain a large number of form-function mappings like the English articles or prepositions. Future research on the Competition Model taking this approach can also look into production data of more controlled elicitation methods. Our sample size is relatively small compared to the usual sample sizes in SEM research. But with free elicitation, we have no control of the sample size for the modeling analysis since it is determined by the number of article cues naturally produced by the learners. Even with more learner texts of the same kind, the type of produced cues remains stable. Controlled elicitations that include more cue types will address the sample size issue for the modeling analysis. In addition, we used existing learner corpus data and sampled English majors and non-English majors for the investigation on levels of L2 competence. We could not obtain direct information from the corpus regarding the learners' bilingual proficiency and other factors such as age of acquisition or amounts of L2 exposure, which have also been shown to be of significant relevance to cue-driven language acquisition in L2 studies of the Competition Model (McDonald, 1987; McDonald and Heilenman, 1991; Sasaki, 1991; Liu et al., 1992; Reyes and Hernandez, 2006). This is a limitation of the current study. Future studies can collect first-hand information from L2 learners for the modeling on the relationships among the variables of interest.

## Conclusion

The current study provides strong statistical modeling evidence for the application of the Competition Model to second language production. College-level Chinese EFL learners' written production of English article cues was heavily influenced by input frequency and followed the Zipfian distribution. The finding that cue reliability was a significant determinant of successful L2 learning for the more competent English majors but not for the less competent non-English majors constitutes strong empirical evidence in support of one of the central claims of the Competition Model. Reliability was identified as the more influential factor when learners progressed to a more advanced stage of cue learning, signaling that learners have developed a more native-like pattern of language use. The methodological innovation of the study generates novel understandings of the Competition Model which creates a new direction to future research on the model. The study also contributes to the recent development of the usage-based approach to second language learning and reveals the rich nature of input-based learning of the English article construction.

## Data Availability Statement

The data analyzed in this study is subject to the following licenses/restrictions: The data of the current study was sampled from the written section in the Spoken and Written English Corpus of Chinese Learners (Version 2.0) (SWECCL) (Wen et al., 2008). The corpus was published in 2008 by the Beijing Foreign Language Teaching and Research Press, and is available for purchase. The corpus is under copyright protection and is not freely available to the public. Requests to access these datasets should be directed to the corresponding author.

## Author Contributions

All authors listed have made a substantial, direct and intellectual contribution to the work, and approved it for publication.

## Conflict of Interest

The authors declare that the research was conducted in the absence of any commercial or financial relationships that could be construed as a potential conflict of interest.

## Footnotes

^1^The distinction between count nouns and mass nouns is not categorical. Some hybrid nouns have the properties and behaviors both as a count noun and a mass noun. Even prototypical count or mass nouns may be used as the other category in some special circumstances.

## Appendix

**Appendix 1:** Essay prompt topics in the learner corpus sample.

**No**	**Topics**
1	Does modern technology make life more convenient, or was life better when technology was simpler? Write an essay to state your own opinion.
2	Education is expensive, but the consequences of a failure to educate, especially in an increasingly globalized world, are even more expensive. Write an essay to state your own opinion.
3	Some people think that famous people are treated unfairly by the media, and they should be given more privacy, while some others think that this is the price of their fame.
4	Some people say the government shouldn't put money on building theaters and sports stadiums; they should spend more money on medical care and education. Do you agree or disagree? State the reasons for your view.
5	Some people think that university education is to prepare students for employment. Others think that it has other functions. Discuss and say what other functions you think it should have.
6	Which skill of English is more important for Chinese learners? Some people think that we should give priority to reading in English, while others think speaking is more important. Write an essay to state your own opinion.
7	Some people think that children should learn to compete, but others think that children should be taught to cooperate. Express some reasons of both views and give your own opinion.
8	Will modern technology, such as the internet ever replace the book or the written word as the main source of information? Write an essay to state your opinion.
9	Nowadays, more and more college students rent apartments and live outside campus. Is it appropriate? State your opinion about this.

**Appendix 2:** Intercorrelation matrix across groups.

	**All**				**English majors**				**Non-English majors**			
	**V1**	**V2**	**V3**	**V4**	**V1**	**V2**	**V3**	**V4**	**V1**	**V2**	**V3**	**V4**
V1	1				1				1			
V2	–0.07	1			–0.05	1			–0.36^^*^^	1		
V3	0.83^**^	0.09	1		0.83^**^	0.11	1		0.75^**^	0.03	1	
V4	0.13	0.18	0.09	1	0.09	0.28	0.08	1	0.06	–0.09	–0.03	1

*V1 = cue availability; V2 = cue reliability; V3 = L2 frequency; V4 = L2 accuracy*.

** *p < 0.01*;

* *p < 0.05*.
